# Simple solutions to wicked problems: Cultivating true believers of anti-vaccine conspiracies during the COVID-19 pandemic

**DOI:** 10.1177/13675494231173536

**Published:** 2023-05-22

**Authors:** Stephanie Alice Baker, Eugene McLaughlin, Chris Rojek

**Affiliations:** City, University of London, UK

**Keywords:** Alternative beliefs, anti-vaccination movement, conspiracy theories, COVID-19 pandemic, disinformation, expertise, medical misinformation, trust, truthers, wicked problem

## Abstract

The pandemic has produced an abundance of medical misinformation, disinformation and conspiracy theories about the safety and efficacy of vaccines. Many of these narratives appear impervious to scientific evidence and indifferent to the authority of the state. This has resulted in ‘true believers’ being cast as paranoid and irrational. In this article, we take a different approach by exploring the cultural appeal of anti-vaccine conspiracy theories about COVID-19. Drawing on qualitative analysis of two leading figures of the anti-vaccination movement – Robert F. Kennedy Jr. and Joseph Mercola – we demonstrate how these influencers establish authority by staging indignation against a corrupt scientific establishment and positioning themselves as Truthers offering simple solutions to complex (wicked) problems. By conceptualising what we refer to as the Truther Playbook, we examine how anti-vaccine Truthers capitalise on existing grievances and conditions of low institutional trust to further solidify people’s troubled relationship with institutional expertise while drawing attention to the structural conditions and social inequalities that facilitate belief in conspiracy theories. We contend that conspiracy theories offer not only offer alternative facts and narratives but are predicated on identification and in-group membership, highlighting the limits of debunking as a strategy to tackle disinformation.

The COVID-19 pandemic has highlighted the public’s fraught relationship with expertise (Nichols, 2017). At the start of the pandemic, prior to the development of safe and effective vaccines, governments around the world experimented with a series of policies designed to limit the spread of the virus. These included public health interventions involving mask mandates, government lockdowns, school closures, quarantine measures and travel restrictions. The public response to government policies has ranged from compliance to denial, often fluctuating somewhere in between, resulting in a series of protests both online and offline. In some cases, these have merged with conspiratorial discourse. It is easy to dismiss the challenge to expertise as misinformation and conspiracism. What these evaluations obscure are the unequal trade-offs people have experienced as a result of these measures: mortality, morbidity, mental illness, unemployment, social isolation and homeschooling, to name a few. COVID denial has emerged in a context of low institutional trust, where those experts ‘led by the science’ have not only got things wrong (e.g. the World Health Organization’s (WHO) initial advice on transmission and mask-wearing), but concealed vital facts from the public (e.g. Dr Fauci’s dismissal of the efficacy of mask-wearing in early 2020 to protect masks for health workers due to supply issues) and in some cases broken the lockdown rules they established to contain the virus (Fancourt et al., 2020). In addition, scientists have not arrived at a consensus on the origins of the virus or the most effective public health measures to respond to the pandemic. This environment of distrust, confusion and conjecture, and the public sentiments of suspicion, anger and doubt they have aroused are not simply emblematic of the failure of expertise, but the complexity of the crisis at hand.

COVID-19 exemplifies what the design theorists Horst Rittel and Melvin Webber term a ‘wicked problem’ (Klasche, 2021; Schiefloe, 2021). A wicked problem is a complex and challenging policy issue devoid of a simple solution. In their discussion of the dilemmas of social planning, Rittel and Webber (1973: 155) noted that ‘the search for scientific bases for confronting problems of social policy is bound to fail, because of the nature of these problems’. Whereas science has developed rigorous methods to test and respond to ‘tame’ problems where there is consensus on how to approach and manage them (e.g. heart surgery), there are no definitive and objective solutions to ‘wicked problems’ (e.g. climate change, poverty). Due to their indeterminacy, wicked problems complicate the linear model of *problem definition* and *problem solution* characteristic of ‘tame’ problems (Buchanan, 1992). They are characterised by disagreements about the definition of problems and their solutions, which are not simply evaluated as true or false, but perceived as good or bad. This moral impulse stems from the fact that in a pluralistic society, policymakers must negotiate between various clients and decision-makers with competing interests, viewpoints and values (Coyne, 2005: 6). In addition to defying objective formulations and solutions, wicked problems produce uncertain and unintended effects. Wicked problems are the norm for policymaking rather than the exception (Coyne, 2005: 12), with COVID-19 presenting policymakers with a wicked problem that defied simple government solutions (Klasche, 2021; Schiefloe, 2021).

Despite claiming to be ‘led by the science’, the pandemic confronted politicians and policymakers with a wicked problem that science alone was unable to answer. While simplistic dichotomies pitching individual liberty against the common good have become standard in hyper-partisan media environments, in the midst of the crisis, trade-offs have been made and some facts and experiences elevated over others. As a result, the harms and benefits engendered by the pandemic have been unequally distributed in society. In this article, we contend not only that the ‘science-led’ approaches used by governments to respond to the pandemic were doomed to fail because they framed COVID-19 as a ‘tame’ problem, which obscured the nature of the wicked problem at hand, we also demonstrate that claims by politicians during the pandemic that their policies were science-led – and subsequently true – undermined their authority enabling influential anti-vaccination activists to capitalise on the crisis of institutional trust that followed. Our analysis focuses on the self-presentation strategies of two prominent members of the ‘Disinformation Dozen’ – Joseph Mercola and Robert F. Kennedy Jr. – demonstrating how these influencers establish authority among the anti-vaccination movement by staging indignation against a corrupt scientific establishment and positioning themselves as Truthers offering simple solutions to complex (wicked) problems. Previous research into these influencers has focused on the messages they use to encourage vaccine refusal: ‘Covid isn’t dangerous; vaccines are dangerous; and mistrust of doctors, scientists and public health authorities’ (Center for Countering Digital Hate (CCDH), 2020: 3). We contend that the conspiracies these influencers disseminate offer not only alternative facts and narratives, but alternative identities and communities. In what follows, we develop a cultural approach to COVID conspiracy theories by situating the appeal of anti-vaccine influencers in terms of four components we refer to as ‘The Truther Playbook’.

## A cultural approach to COVID-19 conspiracies

COVID-19 has produced more than physical contagion. During the pandemic, there has been an abundance of misinformation, disinformation and conspiracy theories circulating online (Birchall and Knight, 2022; Fuchs, 2021). These narratives, many of which appear impervious to scientific evidence and indifferent to the authority of the state, operate beyond the margins of society. Throughout the pandemic, conspiratorial discourse about the origins, transmission and response to the virus has circulated across mainstream social networks (CCDH, 2021), culminating in what the WHO termed an ‘infodemic’: a state wherein the volume of conflicting information available online leaves people unable to determine what to believe and whom to trust. This uncertainty has enabled a series of influencers purporting to expose the *truth* about COVID-19 to sow suspicion about ‘elites’ and the vaccination programmes they endorse (Baker, 2022). These influencers are referred to hereon as ‘Truthers’ given their assertion to provide ‘truthful’ explanations of causes and cures for COVID-19. These range from claims minimising the severity of the virus to outright COVID denial, including conflicting narratives associating COVID-19 vaccines with surveillance (microchips), transhumanism and depopulation made comprehensible by a coherent ideology (Fuchs, 2021).

Conspiracy theories are ‘a way of making sense of current events’ (Butter and Knight, 2020: 1). Although there is no single definition of the term, conspiracy theories tend to be characterised by a dualistic worldview comprised of innocent victims and a small group of nefarious conspirators, who conspire against them (Butter and Knight, 2015). Michael Barkun (2013: 3–4) identifies three characteristics of conspiracy theories: (1) nothing happens by accident, (2) nothing is as it seems and (3) everything is connected. This definition distinguishes conspiracy theories from conspiracies (actual covert plots) on the basis that conspiracy theories seek to impose meaning on the world and ‘give the appearance of order to events’ (Barkun, 2016: 114). Barkun (2013) also notes that conspiracy theories involve, ‘stigmatised knowledge’. In this regard, ‘conspiracy theory’ is not a neutral term. It is a powerful label, which can be used to discredit opponents (Pelkmans and Machold, 2011: 66, 68). The concept implies a demarcation between legitimate, rational knowledge and illegitimate, irrational non-sense, calling into question the ‘sanity’ and ‘credibility of the person making or asserting the proposition’, possibly rendering them dangerous and unlawful (Bjerg and Presskorn-Thygesen, 2017: 138, 154). The use of the term ‘conspiracy theory’, thus, functions as a ‘rhetoric of exclusion’ with explanations described as conspiracy theories excluded as candidates for truth (Husting and Orr, 2007). Given the central role of power in defining what is regarded as a conspiracy theory, a large body of literature defines conspiracy theories in relational terms, as ideas that challenge official explanations and mainstream knowledge (Drążkiewicz and Harambam, 2021; Harambam, 2020; Pelkmans and Machold, 2011).

Prior to the pandemic, conspiracy theories had widespread appeal (Barkun, 2016; Butter and Knight, 2020; Harambam and Aupers, 2017; Knight, 2000; Melley, 2000). Research on conspiracy theories has intensified over the last two decades, broadly falling into three categories of explanation (Harambam, 2020). The first category rests on assertions about the psychological attributes and cognitive capacity of those susceptible to believing in conspiracy theories. Much psychological literature casts those who believe in conspiracy theories as pathological and irrational (Aupers, 2012; Coady, 2007). From this perspective, conspiracy theorists are said to possess certain psychological traits including gullibility (Forgas and Baumeister, 2019), paranoia (Robins and Post, 1997), anxiety (Grzesiak-Feldman, 2013) and delusional ideation (Dagnall et al., 2015). Approaches of this kind are influenced by the early works of Karl Popper (1945) and Richard Hofstadter (1964), presenting conspiracy theories as a ‘crippled epistemology’ (Sunstein and Vermeule, 2008) emanating from paranoid minds; not in the clinical sense where the world is experienced as acting against the individual, but rather a ‘paranoid style’ in which the conspiratorial word is seen to act against a culture or way of life which affects millions of others (Hofstadter, 1964).

At the turn of the millennium, a second category of conspiracy theory research began to challenge these psychopathological approaches by seeking to understand their cultural significance (Butter and Knight, 2020: 31). During this period, a number of scholars contextualised the cultural appeal of conspiracy theories in their capacity to offer comfort and order to those contending with the uncertainties, inequalities and complexities of social life (Dean, 1998; Fenster, 1999; Knight, 2000; Marcus, 1999; Melley, 2000). Conspiracy theories may be factually incorrect, or contain only a kernel of truth, yet they reflect meaningful responses to the experience of certain political, social and cultural conditions (Bjerg and Presskorn-Thygesen, 2017: 140). According to this view, conspiracy theories ‘make sense of the inexplicable, accounting for complex events’ (Melley, 2000: 8), providing a ‘quick-fix’ to complex problems (Knight, 2000: 8). These approaches reject the pathologising tendency of earlier work on the topic, presenting conspiracy theorists’ deep-seated distrust of multinational corporations and the state as a reasonable response to the complexity and opacity of a globalised, capitalist world (Aupers, 2012). A third category builds on these cultural approaches, one which is attuned to the embodied, reflexive and biographical experiences of conspiracy theorists (Harambam, 2020: 131–132). From this perspective, conspiracy theorists are not conceptualised as a homogeneous collective, but a fluid network of different groups, identifying with distinct beliefs, practices and worldviews (Harambam and Aupers, 2017). By seeking to understand the cultural appeal of conspiracy theories rather than debunk, discard or deride those who believe in them, these approaches deviate from the idea that conspiracy theories signify pathology or paranoia (Butter and Knight, 2020: 4; Smith et al., 2022) or are limited to fringe groups (Drążkiewicz and Harambam, 2021: 3).

In this article, we build on this research by exploring the cultural context in which influencers spreading anti-vaccine messaging flourish. Rather than view vaccine conspiracy theories as irrational or a ‘problem of knowledge deficiency’, we concur with previous scholars that part of the appeal of vaccine conspiracy theories is that they affirm disillusionment with the healthcare system and pharmaceutical industry in late capitalism, associating vaccines with power and profit (Drążkiewicz, 2021; Sobo, 2021). The focus of this article is not on those who believe in anti-vaccine conspiracy theories, instead we examine the Playbook prominent influencers use to encourage vaccine refusal. Our aim is not to speculate on the intention of COVID Truthers – some of whom might be genuinely ‘convinced they are helping to reveal the truth’ (Butter and Knight, 2020: 2) compared to others who use the crisis for purely commercial or political ends – but rather to understand their cultural appeal in the context of the pandemic. As such, we contribute to a broader sociological agenda that puts the meaning these forms of knowledge have at the centre of our analysis (see Harambam, 2020: 19–24).

The Internet provides a fertile space for conspiracy theorising to circulate (Baker, 2022). The low barriers to entry enable content creators to produce and share conspiracies online. The infrastructure of social networking sites, which bring disparate users together, creates the conditions for networked disinformation online. The online information ecosystem is especially susceptible to conspiracy theories given the architecture of influence networks and the ways in which users are algorithmically steered towards contrarian, provocative and hateful content. Influencers use platform affordances to target specific demographics and cross pollinate conspiracies to like-minded groups with overlapping interests (Baker and Walsh, 2022). The association between social media, disinformation and conspiracy theories became so intertwined during the pandemic that the CCDH coined the term ‘Disinformation Dozen’, to refer to twelve influencers estimated to be responsible for 65 percent of anti-vaccine content during the COVID-19 pandemic (CCDH, 2021). These anti-vaccine influencers include Joseph Mercola, Robert Kennedy Jr., Ty and Charlene Bollinger, Sheri Tenpenny, Rizza Islam, Rashid Buttar, Erin Elizabeth, Sayer Ji, Kelly Brogan, Christiane Northrup, Ben Tapper and Kevin Jenkins. Members of the ‘Disinformation Dozen’ self-brand in a variety of ways to appeal to specific audiences. Some strategically target women and mothers, while others target groups on the basis of race, religion and politics. What unites these 12 individuals is that they profit by directing consumers to alternative health products and services under the premise of vaccine refusal. They also facilitate strong emotional attachments by subscribing to what we refer to as ‘The Truther Playbook’ (discussed below). In what follows we examine how influencers have elevated themselves as ‘Truthers’ during the pandemic and assemble conspiracy theories that appeal to marginalised and alienated communities.

## Method

In this study, we employ a case study approach to examine the presentation strategies of the two most popular members of the Disinformation Dozen: Joseph Mercola and Robert F. Kennedy Jr., both of whom use online platforms to promote vaccine refusal.

Joseph Mercola is a qualified osteopathic physician and a high-profile wellness entrepreneur. He is a long-standing critic of what he refers to as the ‘medical industrial complex’ – the network of global multinational corporations which conducts research and provides healthcare products and services for a profit. Mercola (2014) presents himself as a debunker, who has broken with the established medical hierarchy and aligns himself with the powerless and the exploited. He has built a career appealing to those disillusioned with mainstream medicine by promoting alternative remedies. During the COVID-19 pandemic, his messaging has extended to attacking official public health directives on lockdowns, vaccines and vaccine passports (Mercola and Cummins, 2021). Mercola is not a disinterested party, since he has substantial commercial interests in propagating his alternative medicine message. His company – Mercola LLC – sells alternative medical treatments and supplements. He is a best-selling author and has been a consistent funder of the National Vaccine Information Center (NVIC), which has been identified as ‘the core of the established anti-vaccine movement’ (CCDH, 2020).

Robert F. Kennedy Jr., a member of a prominent political family in the United States, made his name as a high-profile environmental lawyer and activist. He refers to himself as a heretic who is willing to take on vested interests. Kennedy is not new to conspiracy theories. He popularised the idea that ‘carbon tycoons’, such as the Koch brothers, were conspiring with a network of climate change deniers to wreck any possibility of a new green economy, arguing that laws should be introduced to punish climate change deniers. Since the late 1990s, Kennedy has pursued a high-profile anti-vaccination agenda. During this period, he helped found the *Food Allergy Initiative*, the world’s largest private funding source for food allergy research, and began to proclaim the ills of childhood vaccines. In 2016, he founded the Children’s Health Defense, a public health advocacy organisation dedicated to ending exposure to neurotoxic mercury in fish, medical products, dental amalgams and vaccines. Kennedy (2015) claims to be ‘pro-vaccine’. Despite these disavowals, he has become a leading proponent of the anti-vaccine movement (CCDH, 2021).

These influencers were selected on the basis of their authority among the anti-vaccination movement (online followings, engagement, growth), and the profit their companies accrued during the pandemic (CCDH, 2021). Mercola, who claims his personal website is the #1 Natural Health Website, was described by the *New York Times* in 2021 as, ‘The Most Influential Spreader of Coronavirus Misinformation Online’ (Frenkel, 2021). Together with Mercola, Kennedy has become the figurehead of one of the most popular alternative and natural medicine sites in the world, the *Children’s Health Defense*. Since the start of the pandemic Kennedy’s organisation has expanded its global reach, hiring staff in Canada, Europe and Australia and translating articles into French, German, Italian and Spanish. The organisation draws millions of monthly visitors to its website and, despite being pitched as a non-profit organisation, doubled its profits during the first year of the pandemic with *Associated Press* reporting that the charity filed revenue of $6.8 million in 2020 (Smith, 2021). In light of their social and economic reach, this article seeks to understand the appeal of these influencers as trusted alternatives to official public health experts.

Our analysis of Mercola and Kennedy was conducted from 1 January 2021 to 31 May 2022. During this period, we analysed their public social media profiles on Twitter, Facebook and Instagram, as well as their personal websites and newsletters. These private spaces were particularly important for our study given the stricter content moderation policies introduced by tech platforms during the pandemic to combat COVID misinformation, which resulted in the suspension of Kennedy’s Instagram account and Mercola removing content across his social media platforms to avoid ‘censorship’. We also analysed the books and documentaries these influencers published during the pandemic. Mercola’s book, *The Truth About Covid-19: Exposing the Great Reset, Lockdowns, Vaccine Passports, and the New Normal* (2021) was ranked first in Amazon’s Best Seller Rank for ‘Medicine History & Commentary’ when it was published and maintained this rank at the start of 2022. Kennedy’s (2021) book, *The Real Anthony Fauci: Bill Gates, Big Pharma, and the Global War on Democracy and Public Health* was rated second on Amazon’s Best Seller Rank in the ‘Biological Sciences’ and ‘Medicine & Health Care Industry’ sections at the same time. Rather than scrutinise the veracity of the COVID-19 conspiracy theories spread by these influencers, we examine how they present themselves in these public and private spaces. Content was coded using a grounded theory approach. Our analysis identified four interconnected themes: (1) positioning against an evil, corrupt establishment; (2) access to true, ‘stigmatised’ knowledge, (3) calls to action and (4) offers of guidance, which translate into a series of promises that we describe below as ‘The Truther Playbook’.

## Findings: the truther playbook

‘Trutherism’ – or what is also termed the 9/11 truth movement – refers to ‘true believers’ who promote alternative theories for the 9/11 terrorist attacks (Kay, 2011; Richey, 2017). Recent research demonstrates that those who endorse 9/11 Truther conspiracy theories believe that government and public health authorities are lying to the public regarding the effectiveness and safety of vaccinations. Goldberg and Richey (2020) contend that all three conspiracies have a negative correlation with political trust, political knowledge and education and a positive correlation with authoritarianism. The mainstreaming of these conspiracy theories was made possible by the Internet with popular websites and journalists giving fringe beliefs legitimacy (Barkun, 2016). Influential social media users play a crucial role in popularising conspiracy theories with the pandemic giving rise to a new type of COVID Truther focused on discovering the truth about the origins of the virus and the best way to respond to it. It is easy to dismiss the rise of Truthers during the pandemic as mere conspiracy theories. However, what such labels can obscure are the deep needs and wants that Truthers satisfy, and how they articulate the demands of those groups typically marginalised from society. Part of their appeal is to offer ontological security in a world that is experienced by many as disorderly and insecure. In this regard, the ‘Truther Playbook’ markets itself to ‘seekers’ through four tantalising promises cloaked in the language of truth:

### The promise of identity and belonging

COVID Truthers offer followers access to an exclusive in-group identity. A Manichaean belief system casts the world into good and bad actors, and light and dark forces. It is not simply that individuals and institutions have acted through ignorance or error; their opponents are framed through a binary moral outlook as ‘corrupt’ and ‘evil’. Group integration is generated by identifying external foes that purportedly threaten the group’s beliefs and way of life. This theme is strikingly mobilised by COVID Truthers, who contrast the control of elites with the felt powerlessness of ordinary people. The principle of social integration is based on the identification of these dark, self-serving forces – Big Pharma, the ‘Deep State’ and technocratic elites – that are conspiring to victimise ordinary people. There can be no ‘Defender’, as Kennedy refers to himself, without the preceding idea of a dangerous enemy. Unsurprisingly, members of the medical and political establishment – Fauci and Gates – feature as evil elites – Fauci and Gates – in the titles of Kennedy and Mercola’s pandemic publications.

Mercola’s campaign against official public health advice on COVID-19, as well as his anti-vaccination stance, subscribes to the ‘Truther Playbook’. In Mercola’s analysis, Bill Gates emerges as a veritable prince of darkness, intent on transferring data gathered from biometric tracking into a wider business model (Mercola and Cummins, 2021: 32, 43–47). Official diagnosis and public health policy are tainted by allegations of financial gain and the attempt to consolidate dominance. Doctors and health officials are portrayed as corrupt and self-interested, conspiring to use the pandemic for power and profit. Daily newsletters use repetition to support this view, describing ‘How Fauci and Gates Paved the Way for Vaccines’ as part of ‘The Evil Plot to Enslave Humanity’. Mercola’s outlook is predicated on being contrarian and ‘untainted’ by such corruption. Despite being a medical doctor, Mercola presents himself as a Truther, who bluntly dismisses the official narrative of the origins of the virus as a ‘hoax’ (Mercola and Cummins, 2021: 1–32). From his standpoint, the virus certainly originated from a Chinese government scientific laboratory in Wuhan. It was either an accidental leak, or it was deliberately released. A crucial feature of this narrative is the allegation of an elite cover up: ‘The Deadly COVID Lies Officials Keep Repeating’, ‘[COVID-19] The Orchestrated Plan to Rob You of Christmas’. Mercola proposes that the Chinese regime is colluding with interests of Big Pharma, Big Tech, the scientific establishment, Western governments, the World Health Organization and the World Economic Forum (Mercola and Cummins, 2021: 31). The presentation of the global health risks posed by COVID-19 offers interrelated economic and political opportunities. Economically, COVID-19 was seized upon as a Strategic Investment Vehicle for developing new policing mechanisms to regulate behaviour in public places and expand investment in scientific and medical research. Politically, it opens up a new front for governments to violate civic liberties through tracking arrangements and biometric testing data gathering.

Kennedy has similarly been vociferous in arguing that regulatory agencies have been infiltrated and corrupted by multi-national corporations to such an extent that corruption has been normalised. Kennedy (2005) claims that this regulatory capture motivated his ‘reluctant’ involvement with the vaccine debate and his decision to expose the corruption practised by the medical-industrial complex. He alleges that elites control medical research, falsify scientific findings and that regulatory agencies licence vaccines cause a range of developmental health conditions. Much of Kennedy’s early activism focused on the vaccine ingredient, Thimerosal, warning parents and pregnant women of the dangers of the mercury-based additive and campaigning for Thimerosal to be removed from routine paediatric vaccines (Kennedy, 2015). Thimerosal was eventually removed from childhood vaccination programmes in the United States in 2003, only to be reintroduced the following year in the multidose flu vaccine. Kennedy’s book on the neurotoxin alludes to its links with ADD, ADHD, SIDS, and speech and language delays, suggesting that these risks are concealed by authorities due to the major conflict of interest between regulatory agencies and Big Pharma. As a result, the stage was already set for a multitude of global corporate interests to coalesce to reap unprecedented rewards from the ‘manufactured’ crisis that is COVID 19.

During the pandemic, Kennedy’s claims have become more extreme, predicated on his criticism of the medical establishment. Kennedy’s primary villain is Dr Fauci, who he represents as the figurehead of a corrupt medical industrial complex. In his weekly newsletters, Kennedy frequently reminds his followers about the collusion and elite corruption that characterises the medical establishment and pharmaceutical industry. These range from general questions about power – ‘Who’s Running the World?’ – to more extreme allegations of ‘Blood Money’, vaccine injury and death, cloaked in language about evil elites, ‘the technocratic state’ and the medical establishment: ‘Gates-Fauci Formidable Nefarious Partnership + Gaslighting Autism Families’, ‘Evil: ‘The Weaponization of the Medical Establishment’, ‘Global Elites Plan to “master the Future”’, ‘WHO Plotting Global Totalitarian State’. Kennedy’s Instagram account was suspended in 2021 for repeatedly sharing such views.

Following Kennedy’s suspension from Instagram and Facebook in 2022 for promoting medical misinformation, most of his messaging has been communicated via the *Children’s Health Defense* and their weekly newsletters. The organisation describes its mission:

To end the childhood health epidemics by working aggressively to eliminate harmful exposures, hold those responsible accountable, and establish safeguards so this never happens again.

What is evident from this mission statement is how Kennedy’s organisation is framed in opposition to corporate and government interests. His website reinforces this messaging, describing Kennedy as ‘The Defender’ to an array of corporate adversaries: Big Pharma, Big Energy, Big Food, Big Tech and Big Chemical. In 2021, the organisation perpetuated the ‘great reset’ conspiracy theory, claiming that ‘global elites’ will use the pandemic to advance their interests.

The observation that conspiracy theorists appeal to totalising narratives of good and evil is well documented. Conspiracy theories are often characterised by the belief that powerful elites control (or seek to control) society and politics (Fenster, 1999), providing a vital component of the conspiritualist movements that have proliferated throughout the pandemic (Baker, 2022). However, unlike the notion of ‘conspirituality’ conceptualised by Ward and Voas (2011), the Truthers examined in this article do not subscribe to the New Age vision that evil will be eclipsed by spiritual salvation. Instead, fears about the toxins and chemicals found in vaccines are used to sell alternative products and services: books, docuseries, courses, subscriptions and supplements as sources of redemption. Their presentational style resonates with the observations of those scholars who refrain from such essentialist definitions of conspiracy theories, defining conspiracy theories in relational terms, as sets of ideas that challenge mainstream knowledge and officially sanctioned truths (Birchall, 2006; Pelkmans and Machold, 2011).

### The promise of true knowledge and enlightenment

COVID Truthers promote themselves as modern ‘prophets’ and ‘defenders’ of the people, purporting not only to protect the vulnerable but also to have access to a privileged epistemology. They respond to those disillusioned with the government’s official narrative – ‘The Deadly COVID Lies Officials Keep Repeating’ (Mercola) – by proclaiming to reveal alternative facts. Alternative facts are not simply counter to the mainstream; they are allegedly hidden and deliberately concealed from the public, often constituting what Barkun (2016) refers to as ‘stigmatised knowledge’. The notion that COVID Truthers possess a privileged epistemology is communicated through the promise to ‘expose’, ‘release’ and ‘reveal’ the Truth, as exemplified by the title of Mercola’s bestselling book: *The Truth About Covid-19: Exposing the Great Reset, Lockdowns, Vaccine Passports, and the New Normal* (2021).

For Mercola, COVID-19 is being used by elites to advance and consolidate what he calls ‘The Great Reset’: ‘a long term plan to disempower and disenfranchise all but the wealthiest by monitoring and controlling the world through technical surveillance’ (Mercola and Cummins, 2021: 47). Mercola’s analysis operates on the basis of calculated resonance with popular conceptions of being deceived by plutocrats that control the corporate-state axis. To build his case, he utilises the familiar device of challenging official statistics on the relationship between COVID-19, hospitalisation and mortality. He contends that only 6 percent of COVID-19-related deaths in the United States list the virus as the sole cause of death on the death certificate (Mercola and Cummins, 2021: 53). The object of challenging official statistics is to relocate COVID-19 within the range of risk from ‘normal’ bacterial infections. According to Mercola, except for people over 60, the mortality rate from COVID-19 is significantly lower than the conventional mortality rate for influenza (Mercola and Cummins, 2021: 55). Sepsis and co-morbidities, such as obesity, heart disease, and diabetes, are the decisive factors in most deaths that are classified as ‘COVID-related’. At the same time, Mercola deploys statistics in his newsletters to verify his claims – ‘Predicted Vax Statistics Come True, Grim Beyond Belief’; ‘COVID Infection Rate Higher Among UK’s Fully Vaxxed’ – with questioning employed as a familiar technique to sow doubt about the safety and efficacy of COVID-19 vaccines: ‘Deleted Data, How Many Vaccine Deaths are they Hiding?’; ‘What’s Pushing Autism Rates Sky High?’; ‘Are COVID Shots Prolonging the Pandemic?’; ‘Are the Jabs Making People More Prone to Omicron?’; ‘The COVID Jabs – Causing More Harm Than Good?’.

Mercola’s solution is unsurprising – ‘boost’ your immune system by purchasing his supplements which, according to his scientific sources, are safer and more effective than vaccines. Mercola’s views are appealing to those seeking bodily autonomy and for whom COVID is experienced physically as ‘just like the flu’ or what one of his newsletters refers to as ‘Just a Hyped-Up Cold’. What binds these various claims is the idea that the public are being lied to – ‘The Biggest COVID Lies of the Last 2 Years’ – and truth is being ‘censored’; hence claims in Mercola’s newsletters about exposing censored claims, which range from the mundane – ‘The Censored Library Returns: Never Miss Another Article’, ‘The Censored Interview You Must See to Understand COVID’, ‘Smoking Gun Reveals Who’s Really Waging War Against Science’ – to the extreme: ‘Nurse Exposes Hidden Vax Deaths, behind Closed Doors’, ‘The Who’s Who of the Great Reset Plan’, ‘The Reckless Plan Towards Transhumanism’.

Truth also features prominently in the mission statement of Kennedy’s organisation, notably in terms of advocating for ‘transparent government’, exposing elite corruption and protecting children from harm. In an interview featured on his orgnaisation’s website, he describes his aim to ‘expose the truth about vaccine injury and win justice for injured children’. While most of Kennedy’s early anti-vaccine messaging focused on protecting pregnant women and children from harmful ingredients in vaccines, during the pandemic he has shifted towards the topic of medical racism. In March 2021, Kennedy released a video: ‘Medical Racism: The New Apartheid’. The film explores the issue of vaccine safety, reviving former claims of an association between the measles, mumps and rubella (MMR) vaccine and autism, which the film alleges disproportionately harms African American boys, especially when taken below the age of 3, as recommended by the CDC: ‘the CDC data showed black boys who got the MMR on time were 336% as likely to be diagnosed with autism as black boys who waited’. This effect was purportedly ‘not seen in white children’, with the narrator claiming ‘no one vaccine fits everybody. We are not all genetically the same’. The film focuses on the experiences of African Americans, especially the Minneapolis Somali community, where there are disproportionately high levels of autism. The film relies heavily on personal anecdotes as evidence, with parents reflecting on their children’s experiences of vaccine injury. These stories are coupled with experts sharing claims of medical experimentation: vaccine testing in Zimbabwe and Nigeria, and forced sterilisation in Namibia during the slave trade to prevent mixed-race marriages, contributing to the narrative that State and Big Pharma cannot be trusted. The narrator goes on to explain that ‘in 2014, the WHO gave 1 million Kenyan women of childbearing years a tetanus vaccine that contained a secret ingredient used to make women infertile’. Another female reporter interviewed in the film echoes this view explaining that experts believed ‘the vaccine should actually be tested in Africa since it’s easier and people are poorer it’s-it would just be easier for them, and it would be cheaper’ (Children’s Health Defense, 2021).

In proclaiming itself to be ‘exposing the Truth Behind Systematic Racism in Medicine’, the film draws on grievances that African American communities have with the medical establishment, following systematic ethical failures in medicine. These include Dr. Marion Sims’ gynaecological surgical experiments on black enslaved women and the Tuskegee Study conducted by the United States Public Health Service and the CDC between 1932 and 1972, which left hundreds of African American males with untreated syphilis. Consequently, race features significantly in Kennedy’s discourse, appealing to those groups who understandably have developed a fraught relationship with expertise, distrustful of the government and medical authorities. Claims of this kind also draw on anti-government sentiment in the wake of the murder of George Floyd in May 2020, with Kennedy using the #BLM hashtags on his social media posts to frame the film under the guise of racial justice. The distrust that racial minorities feel towards government authorities, as a result of a history of medical discrimination and pervasive racial injustice that persists today, is used to promote vaccine hesitancy and vaccination – or more specifically, ‘unvaccination’ – as a form of racial segregation and discrimination, which Kennedy refers to as ‘the new apartheid’.

Hence, although the victims Kennedy alleges he protects may change, his authority largely rests on the notion of accessing a privileged epistemology. This claim is articulated on three fronts: (1) the idea of an elite cover-up – ‘Censorship just took another deadly turn’, ‘Censorship Attack’, (2) revealing these ‘dark truths’ – ‘Cardiologist Truthbombs’, ‘The “Lie of the Century” will be revealed to the masses’, ‘The Big Reveal is Coming . . .’, ‘Dark Truth About Vaccine Injury Court’, ‘CDC Admits It Didn’t Monitor COVID Vax Injuries’ and (3) sharing access to this secret knowledge; in his own words, ‘applying his tenacious energies and sophisticated strategies to exposing fraud and corruption within the CDC and the pharmaceutical industry’. For example, warning of the nefarious intentions of corporate elites and the untold risks of the novel coronavirus vaccines as exemplified by Kennedy’s new book, *The Real Anthony Fauci* (2021). This insight, which often draws on genuine grievances, is the passport to enlightenment. In this regard, despite claiming to promote ‘rigorous science’, Kennedy’s epistemic absolutism contrasts sharply with the scientific method, which encourages scepticism and doubt.

### The promise of meaning and purpose

Despite their reliance on alternative evidence, part of the appeal of COVID Truthers is the way in which they provide their followers with meaning and purpose. Truthers give their followers a reason to believe in something other than facts and materialism. Belief may take the form of self-improvement, in which personal salvation is communicated through New Age discourse as part of a ‘Great Awakening’, consisting of a utopian vision of a better society (see Ward and Voas, 2011) or a more secular commitment to truth, freedom and justice (Baker, 2022).

Both Kennedy and Mercola urge their followers to join their movements. Kennedy’s followers are encouraged to campaign for justice for the victims of the crimes of the ‘medical-industrial complex’; including children and marginalised groups. Mercola subscribes to a more individualist ethos, which frames mandatory vaccination as an infringement of individual liberties. In his generic wellness posts on social media, followers are assured that they will learn how to ‘take control of their health’ through making informed choices: ‘6 COVID Superstars Finally Endorsed by Government’, ‘What You Need to Know About Early Treatment for COVID’, ‘The Ultimate COVID “Whipping” Toolbox, 22 Tools for 2022’. His personal newsletters promise a deeper commitment to meaning and purpose. While Mercola is not a New Age conspiritualist promising spiritual salvation, there are themes of awakening and claims that breaking free of the ‘matrix’ will result in Enlightenment: ‘Wake up, the “Boosters” Are a Trap (As Predicted)’.

Kennedy similarly presents vaccine mandates as a violation of constitutional rights and liberties. This message is expressed in his newsletters and on social media with posts questioning how vaccine mandates undermine their freedom: through ‘Vaccine Mandates: An Erosion of Civil Rights?’ Kennedy situates the anti-vaccine movement’s opposition to vaccine mandates in a broader civil rights agenda, championing the rights of African Americans to exercise the right to choose what to do with their body and the bodies of their children. In this regard, Kennedy compares racial segregation to non-vaccination and legitimises his authority in relation to his family’s involvement in championing racial justice. Kennedy’s uncle John, and his father Robert, had a celebrated history in the civil rights movement, working with Martin Luther King. His aunt, Eunice, whom he mentions in the film, was a philanthropist, founding the Special Olympics for persons with physical and intellectual disabilities. His uncle Senator Ted Kennedy led the congressional hearings that exposed the racist ‘Tuskegee syphilis experiment’ orchestrated by the US government. By association, Kennedy positions himself as continuing this honourable heritage. In addition to criticising vaccine mandates, he attacks social distancing measures and travel restrictions as unacceptable infringements upon individual liberty. The film concludes with Kennedy making a personal request for donations from viewers, explaining:

Your donation will go towards promoting Medical Racism: The New Apartheid documentary film and protect the health of children. Together we can work towards ending racism in medicine as well as targeting people of color in medical experimentation.

Kennedy’s self-presentation as a Truther is central to his messaging. In a direct call to action, his December 2021 newsletter requests that his followers ‘Plant Seeds of Truth!’ by participating in his new sticker campaign, ‘Stick to the Truth’, which emphasises medical freedom and parental rights. Similar to Mercola, Kennedy’s mission is framed in a broader narrative of distrust of government institutions and corporate elites. However, whereas Mercola’s health solutions appeal to those distrustful of mainstream medicine, Kennedy’s messaging targets those groups perceived as particularly vulnerable to scientific and government corruption: children, women and racial minorities. Kennedy frequently deploys the language of social justice in his social media posts and newsletters as a rallying call to unite his followers. Followers are urged to ‘Unite to Create a Better World’ and reminded about the importance of ‘Seeking Justice and Spreading the Truth’, through explicit analogies to the Civil Rights Movement: ‘We won a revolution before. . .we can win it again’. By positioning himself as a literal ‘defender’ of the public, Kennedy weaponises a movement designed to empower marginalised groups and by appealing to those statistically at most risk from the virus.

### The promise of leadership and guidance

Part of the appeal of Truthers is to proffer order and ontological security in a world that is observed and experienced as disorderly and insecure. In doing so, they speak to the institutional distrust many people feel towards ‘the establishment’ (Baker and Rojek, 2019; Drążkiewicz, 2021; Sobo, 2021). Truthers contrast the power of elites with the felt powerlessness of ordinary people. These claims extend beyond distrust of ‘the establishment’, to the depiction of elites as conspiring to harm ordinary people: ‘Are the Un-Jabbed Headed to a Quarantine Camp in 2022?’, asks Mercola. The result of this symbolic positioning is not only a rejection of elites and institutional expertise, but the search for alternative leaders to guide individuals towards the pursuit of *truth*. In our previous work we referred to this condition as ‘low trust society’ (Baker and Rojek, 2019). What we meant by this term was not the absence of trust from the current political climate, but rather its transference from traditional professional and technocratic institutions to alternative conduits of knowledge.

Truthers are true believers who seek to encourage others to join their movement. The anti-vaccination movement is often pejoratively described as a cult (Mylan and Hardman, 2021). Whereas traditional cults typically had a single leader, decentralised online social networks have given rise to leaderless social movements. There is no single leader of the vaccination resistance movement. Instead, there are a series of Truthers, such as Mercola and Kennedy, who collaborate online via cross-promotion and affiliate marketing schemes to recruit members to the vaccine resistance movement and encourage vaccine refusal by sowing distrust of medical authorities, and fear and uncertainty around vaccines (CCDH, 2021). The rise of user-generated content enables ordinary users to contribute to the network by asking questions, sharing posts and appropriating anti-vaccination memes (Baker and Walsh, 2022), as exemplified by Kennedy’s initiative described above. In the current information ecosystem, anti-vaccine content is networked online through a variety of different users and platforms, with Truthers emerging as influential nodes in online social networks.

Truthers develop rituals of behaviour to cement membership and social integration. Group sentiments are established and reinforced by weekly newsletters and daily social media posts. These frequent interactions – typically communicated via close ups of the Truther in the style of an intimate friend – contribute to the development of parasocial relationships (Horton and Wohl, 1956). These parasocial interactions tend to be more intense than those experienced via the mass media (e.g. television, radio) because social media offers the potential for *direct* communication with the influencer whether realised or not (Baker and Rojek, 2019). Despite their parallels with the countercultural movements of the 1960s, rather than collectively seek social and political change, many Truthers build online communities and monetise their solutions. Hence, amid Mercola’s more extreme claims of an elite cover-up and ‘The Great Reset’, there are frequent reminders that his followers are customers: ‘Valued Customer, thank you for letting me join your health journey’. His daily newsletters provide direct links to his personal website where his followers can purchase supplements, protein powder, pet food, clothing and a range of home ‘essentials’ to optimise their health.

## You are not alone: why the Truther Playbook matters

COVID-19 has multiplied platform labour dedicated to disseminating a multitude of conspiracy theories about the virus. Distrust of established expertise and elites fuels the creation of alternative explanations, which bear no relationship to approved scientific methods of validity testing. As noted above, the Covid Truthers who have come to the fore during the pandemic adhere to a Playbook that resonates with personal vulnerabilities and exposes the contradictions in normative scientific practice to claim the mantel of science for themselves. In so far as science follows Popper’s (2004 [1963]) approach, in which all propositions and data are subject to repeated testing and defined as potentially refutable, it is ill-suited to ‘wicked problems’ such as COVID-19. The Popperian emphasis on falsifiability rather than verifiability as the aim of the scientific enterprise, effectively puts all scientific claims under permanent risk of de-legitimation. During the pandemic, governments have gone to great lengths to maintain that their COVID-19 policies are ‘led by the science’. The problem is that ‘the science’ has not achieved consensus about the correct programme of intervention, as alluded to with debates regarding origin, transmission and treatments for the virus. There have been disputes about the necessity of wearing masks at all times, the correct measures – quantified in inches, feet or metres of social distancing – and the efficacy of vaccines. Part of this ambiguity is because scientific knowledge production is uncertain, unsettled and evolving (Latour, 1987; Wolpert, 1993). The pandemic also raises moral and political questions that science is unable to answer, facilitating spaces in which Truthers can establish counter-narratives by exposing the fallibility of official solutions to these ‘wicked problems’.

The proliferation of COVID conspiracy theories is a world away from the rational-communicative model that Habermas (1962) believed would sustain consensus in postwar democracies. Habermas developed his position from a critical engagement with the first Frankfurt School argument of ‘the eclipse of reason’ (Horkheimer, 2004). That is, the proposition that *reason* elevates utility, in the form of self-preservation, and the domination of nature, as determinate in human agency. In doing so, it relinquishes its critical edge and becomes instead an instrument of control (Adorno and Horkheimer, 1979). Habermas is committed to a planning model of inter-subjective rational linguistics around validity testing. Through the entwinement of rational argumentation, in concrete material settings, speech and action are capable of producing the affirmative reconciliation of different interests and consensual agreements. However, if one adds Popper’s falsification principle to this, it must be the case that all consensual agreements so composed are subject to ‘a just in time’ principle. Whatever rational deliberation can achieve, in the shape of discursive rationality, is provisional. In so far as Popper and Habermas contribute to the theory of modernisation, they run counter to any aspiration that reason and science will culminate in a consensual, settled form. Instead, they think of modernity as a condition in which observation and experience are perpetually in a state of suspension wherein obedience and scepticism go together hand in hand. This is a great advance on charismatically or traditionally legitimated cultures, which portrayed life as ordained by immoveable social hierarchies or pre-ordained by divine privilege (Weber, 1968). It also comes with problems of its own making. ‘Life in suspense’ means that everyday experience must put up with thin levels of consensus about the nature of social reality, social purpose and common social interests. The thinness of consensus provides a frail foothold against the eruption of conspiratorial thinking, from which Truthers draw.

This thinness of consensus also explains why attempts to ‘debunk’ COVID Truthers, such as Mercola and Kennedy, have had limited success. Some scholars perceive debunking to be an unsuccessful strategy because it presumes ‘that scholars (can) actually know the real truth, because taking sides in societal knowledge contestations is not what we should do, and because providing more or ‘correct’ information will not even work since knowledge acceptance is dependent on people’s worldviews and identification processes’ (Harambam, 2021: 112–113). While we contend that vaccine conspiracy theories deserve scholarly critique because they can engender imminent physical harm, when debunking takes the form of mockery it can have unintended consequences by fuelling polarisation, strengthening in-group dynamics and giving legitimacy to conspiratorial narratives (Baker and Maddox, 2022). The CCDH’s report on Mercola and Kennedy as the most influential disinformation producers – and the public ridicule that followed – has only increased their notoriety. Harambam (2021) proposes ‘deliberative citizen knowledge platforms’, which assess the quality of information in the public domain in organised cooperation with relevant stakeholders and experts, as a more effective, democratic alternative to debunking conspiracy theories. Future research could explore the effectiveness of deliberative citizen knowledge platforms as a way to respond to anti-vaccination conspiracy theories. Moreover, given that our analysis is limited to examining two prominent COVID Truthers in the context of the pandemic, future research could explore how other influencers use the Truther Playbook to disseminate conspiracy theories, and with what effect.

## Conclusion

The extraordinary expansion and acceleration of the conspiracy milieu during the COVID-19 pandemic offers an unexpected lesson for sociologists. The modernisation thesis proposed that reason and science would inevitably leave the values, beliefs and practices of traditional society behind. In particular, organised religion as a key placeholder of the traditional milieu would be replaced by the secularism and cognitive rationalism provided by scientists. Habermas (2008), the architect of the defence of reason through communicative competence in postwar democratic thought, now uses the term ‘post secular’ to refer to the ‘unexhausted force’ of non-scientific belief systems in contemporary society. The flourishing of conspiracy theories exposes the hyperbole embedded in modernisation theory (Aupers, 2012; Harambam and Aupers, 2017; Ward and Voas, 2011). Conspiracy theories, spread by COVID Truthers such as Mercola and Kennedy, convey the profound attraction of alternative anti-establishment forms of explanation, not simply as facts, but as precursors to in-group membership, as we have demonstrated through ‘The Truther Playbook’.

Despite their variation, COVID Truthers appeal to their followers by promoting their agenda in a parable of good and evil to further sow distrust of experts and elites and to amplify doubts about the safety and efficacy of vaccines. Truthers foster in-group dynamics by drawing on the grievances of those who feel alienated and disempowered, giving adherents a sense of meaning, purpose, identity and belonging through the promise of a superior epistemology and ontology. The theories Truthers disseminate are contingent on meaning and identification and consequently their knowledge claims elude the scientific falsifiability principle. Moreover, in the context of the COVID-19 pandemic where ‘science-led’ approaches could not account for the complexity of the wicked problem, Truthers simply needed to question the limits of the official narrative–rather than verify their claims–to cultivate true believers.

Gramsci’s (1988) conceptualisation of folklore provides a useful lens through which to understand how the Truther Playbook operates today, and why conspiracy theories have been so prevalent during the COVID-19 pandemic. For Gramsci (1988), folklore stands in an antagonistic relationship to official conceptions of the world, foregrounding ‘common sense’. It is not an elaborated or coherent set of concepts. Rather, it is ‘many sided’ in the sense of including the juxtaposition of different and often contradictory elements and also in that it embraces a continuum of popular knowledge from ‘the crude’ to ‘the less crude’ (Gramsci, 1988: 360). Folklore is built around a religion and morality of ‘the people’, and in certain instances epitomises ‘good sense’. It is flexible, including elements from the past (that are often ‘conservative’ and ‘reactionary’), but also new components which arise spontaneously by ‘forms and conditions of life which are in the process of developing’ (Gramsci, 1988: 161). Gramsci contends that this is why folklore is a ‘much stronger, more tenacious and more effective’ way of making common sense than official scientific culture. This is contrary to modernisation theory. For it demonstrates that modernisation has not rooted out folklore, but rather that folkloric popular thought has adapted to develop an organic relationship with modernisation. These reactions strongly suggest that the civil incorporation of people into scientised, rationalised modernity has limits that have not been fully recognised, while illustrating the depth of distrust with established institutions and elites, especially with the power of the medical-scientific ‘corporatocracy’. If any silver linings are to be found in the official management of wellbeing in a post-COVID world, this may be one of them.
